# Drugs and Bugs: The Gut-Brain Axis and Substance Use Disorders

**DOI:** 10.1007/s11481-021-10022-7

**Published:** 2021-10-25

**Authors:** Sierra Simpson, Rio Mclellan, Emma Wellmeyer, Frederic Matalon, Olivier George

**Affiliations:** Department of Psychiatry, University of California San Diego, La Jolla, San Diego, CA 92093 US

**Keywords:** Addiction, Alcohol, Cocaine, Gut-brain Axis, Metabolites, Microbiome, Opioids, Methamphetamine, Psychedelics, Cannabis

## Abstract

Substance use disorders (SUDs) represent a significant public health crisis. Worldwide, 5.4% of the global disease burden is attributed to SUDs and alcohol use, and many more use psychoactive substances recreationally. Often associated with comorbidities, SUDs result in changes to both brain function and physiological responses. Mounting evidence calls for a precision approach for the treatment and diagnosis of SUDs, and the gut microbiome is emerging as a contributor to such disorders. Over the last few centuries, modern lifestyles, diets, and medical care have altered the health of the microbes that live in and on our bodies; as we develop, our diets and lifestyle dictate which microbes flourish and which microbes vanish. An increase in antibiotic treatments, with many antibiotic interventions occurring early in life during the microbiome's normal development, transforms developing microbial communities. Links have been made between the microbiome and SUDs, and the microbiome and conditions that are often comorbid with SUDs such as anxiety, depression, pain, and stress. A better understanding of the mechanisms influencing behavioral changes and drug use is critical in developing novel treatments for SUDSs. Targeting the microbiome as a therapeutic and diagnostic tool is a promising avenue of exploration. This review will provide an overview of the role of the gut-brain axis in a wide range of SUDs, discuss host and microbe pathways that mediate changes in the brain’s response to drugs, and the microbes and related metabolites that impact behavior and health within the gut-brain axis.

**Graphic Abstract:**

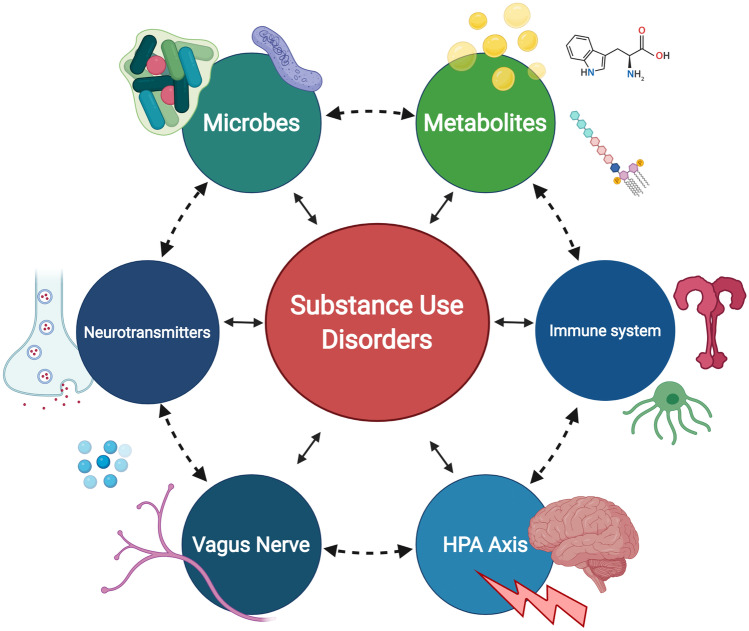

## Gut-brain Axis and Addiction

Despite the impact of substance use disorders on society, few treatment options are effective, underscoring the need to explore novel therapies (Volkow and Boyle 2018). Emerging evidence supports the role of the gut-brain axis in regulating behavior and responses to drugs, and in a larger context, reward and satiety (Han et al. 2018; Van Oudenhove 2014; Bliss and Whiteside 2018). The gut and brain are physically connected through the vagus nerve and chemically connected through metabolites, hormones, and neurotransmitters (Cryan and Dinan 2012; Brookes et al. 2013; Forsythe et al. 2016; Sarkar et al. 2016). The presence or absence of specific microbes modulates the immune system (Belkaid and Hand 2014; Zhao and Elson 2018; Lazar et al. 2018; Gensollen et al. 2016) and regulates inflammation (Lobionda et al. 2019; Blander et al. 2017; Clemente et al. 2018; Tilg et al. 2020). Studies in both humans and preclinical models have demonstrated the critical role of gut microbes in brain function (Mohajeri et al. 2018), mood (Huang et al. 2019), and behavior (Marchesi 2016; Li 2008).

Drugs of abuse are known to alter the composition of the gut microbiome (Mutlu et al. 2012; Bode et al. 1984; Bjorkhaug 2019; Kang et al. 2017; Wang and Roy 2017). Opioids cause constipation by activating µ-opioid receptors in the gut, lengthening the transit time of gut contents (Khansari et al. 2013; Wood and Galligan 2004). Long-term alcohol abuse leads to chronic liver disease (Osna et al. 2017), changes in metabolism (de Timary et al. 2012), bile acid availability (Monroe et al. 1981; Ridlon et al. 2015), and intestinal permeability Kakiyama et al. (2014). Individuals that abuse substances also have distinct changes to dietary preferences. Those with alcohol use disorder (AUD) consume many of their calories through alcohol (Lieber 2003), opioid users increase intake of foods high in sugar (Mysels and Sullivan 2010), many stimulant, psychedelic, and nicotine users reduce food intake (Mineur et al. 2011; Mitchell and Roseberry 2019), and THC initiates binge-like feeding behavior (DiPatrizio et al. 2011). The substitution of dietary choices and changes in behavior only compound drug-related fluctuations in the gut microbiome but also extend to the oral microbiome. For example, smoking cannabis or nicotine can cause degradation to the oral epithelium, the acidic nature of cocaine can be detrimental to jaw integrity, and alcoholic drinks are acidic and alcohol itself can kill oral microbes. Over an extended time, oral dysbiosis and ingestion of increased opportunistic and pathogenic microbes that reside in the mouth may have negative consequences on gut health long-term (Olsen and Yamazaki 2019).

**Fig. 1:**
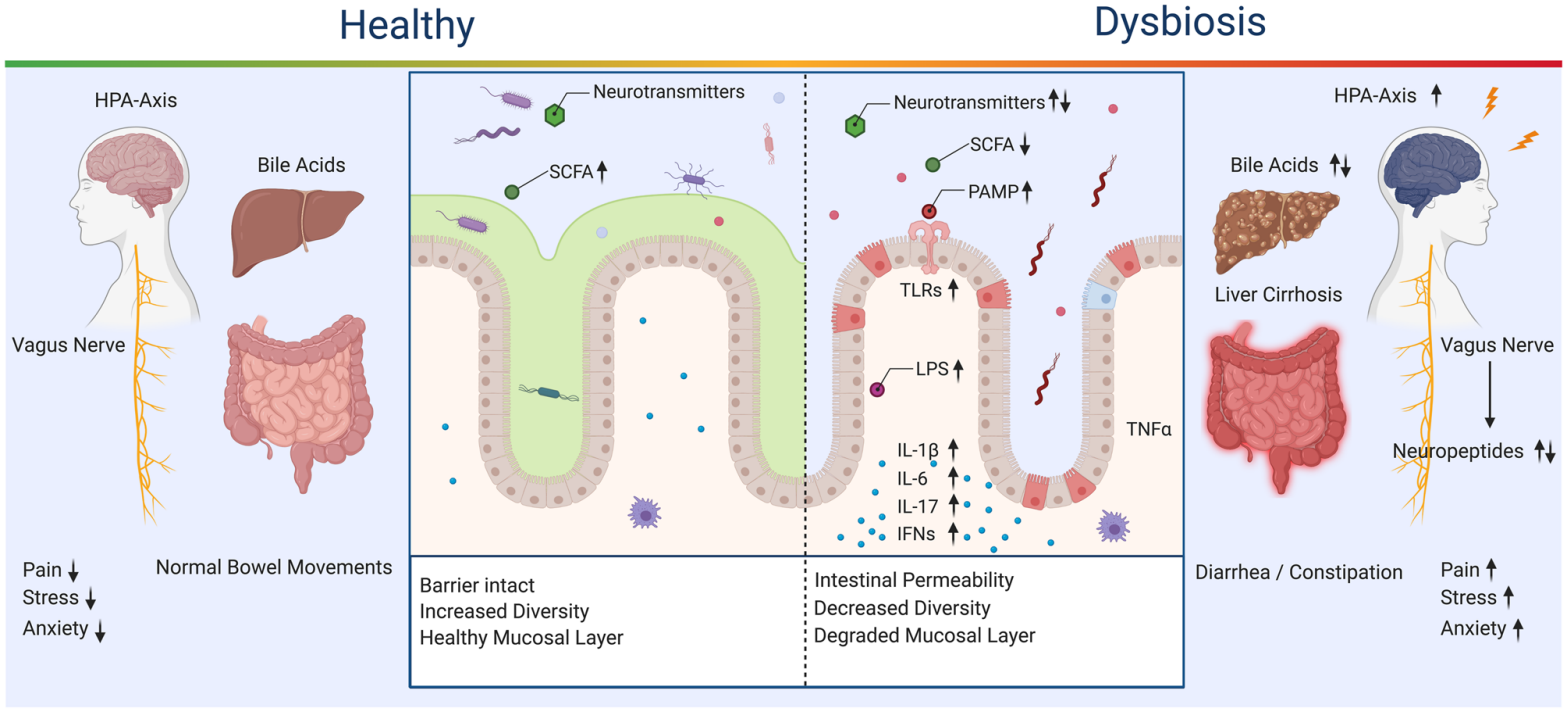
**Healthy and Dysbiosis States of the Gut-Brain Axis.** Dysregulation of the gut-brain axis results in alterations to available microbes, metabolites, and inflammatory signals. SCFA, bile acids, and neurotransmitters are decreased or dysregulated through community shifts. A decrease in SCFA impairs tight junctions and allows for intestinal permeability, which is linked to activation of a wide range of proinflammatory signals and pathways. Bile acid dysregulation contributes to liver cirrhosis and alterations of the microbial community through the antimicrobial properties of bile acids. Inflammation in the gut is increased through the hypothalamic pituitary axis (HPA) and has feedback to the central nervous system, increasing pain, stress, and anxiety

The gut-brain axis represents a highly integrated, dynamic ecosystem, with both central and peripheral mechanisms playing a role in balancing the microbial communities and behavioral feedback mechanisms for optimal function (Fig. 1). Due to the limited efficacy of the treatments for substance use disorders, there is a need to understand better the potential for the gut-brain axis to regulate behavioral responses to drugs and other contributing comorbid neurological states. A comprehensive cross-sectional analysis of the literature is currently missing—this review fills that gap by exploring how substance use disorders both influence and are influenced by the microbiome. First, we will discuss several critical microbiome and gut-brain axis elements including communication pathways, such as the vagus nerve, microbial metabolites, and immune system dysregulation. Then we will discuss the impact of specific drugs on the microbiome and gut-brain axis. Although there is limited literature on this topic for some drugs of abuse, we will include those that have been widely studied (alcohol, psychostimulants) as well as those that are emerging (nicotine, opioids, psychedelics, and cannabis).

## Critical Elements of the Gut Microbiome

A healthy microbiome acts as a barrier to preventing the overgrowth of exogenous bacteria and commensal microbes that can potentially turn pathogenic (DeGruttola et al. 2016; Bien et al. 2013). The modern western diet Zinocker and Lindseth  (2018), the overuse of antibiotics (Dethlefsen et al. 2008), and drug use all have a detrimental influence on gut microbiome composition. Processed foods lower the threshold to nutrient absorption (Spreadbury 2012), creating an environment where the colonization of unfavorable bacteria can occur rapidly Stecher (2015). Microbes can swiftly adjust community makeup and metabolism depending on the nutrient supply and composition of surrounding communities (David et al. 2014; Jumpertz et al. 2011; Turnbaugh et al. 2009). While there is no perfect “healthy microbiome,” a diverse diet high in fiber and low in processed foods is beneficial to maintaining a microbiome with increased diversity and resiliency Hills et al. (2019). A resilient microbiome can better adapt to shifts in community makeup and reduce the probability of an imbalance of the natural flora in the gut (dysbiosis) (Stecher et al. 2013; Lozupone et al. 2012; Fassarella 2020). The gut microbiome also participates in the education of the immune system to avoid hyperreactivity to commensal microbes and food antigens. This process occurs through the activation of inflammatory responses through metabolites produced by the microbiome, and the microbial maintenance of the gut epithelium (Brandtzaeg 2011; Hooper et al. 2012; McFall-Ngai 2007; Maynard et al. 2012). If the microbiome is not in balance, the resulting dysbiosis leads to degradation of the gut-epithelium, translocation of microbes, microbial epitopes such as lipopolysaccharides (LPS), and a plethora of metabolites that alter the immune response and signaling throughout the body and brain (Levy et al. 2017). Communication between the gut and brain is not only through indirect pathways like microbial metabolites, but also via the vagus nerve, which is the primary direct pathway of the gut-brain axis (Fülling et al. 2019; Bonaz et al. 2018).

## Vagal Pathways and the Microbiome

One critical avenue for drug-induced alterations of the gut-brain axis is via the vagus nerve, a wandering behemoth of sensory and motor fibers that represents a collection of functionally and structurally diverse connections between the viscera and the brain (Johnson and Wilson 2018). Vagal pathways have been central to exploring the gut-brain axis as a direct connection between the gut and the brain and are thoroughly reviewed elsewhere (Fülling et al. 2019; Bonaz et al. 2018; Breit et al. 2018; Forsythe et al. 2014; Cryan 2019). Vagal afferent terminals are located beneath the gut epithelium, receive signals produced by the gut microbiota (Cawthon and de La Serre 2018). A wide variety of receptors on vagal afferents has been hypothesized to provide a polymodal response to a broad assortment of chemical, mechanical, and hormonal signals (Egerod et al. 2018). Additionally, the vagus regulates immune responses in the gut via the release of acetylcholine, which attenuates immune cell activation through ⍺-7 nicotinic acetylcholine receptors (a7nACHr) (Matteoli and Boeckxstaens 2013; Wang et al. 2003). The vagus nerve is also implicated in behavioral responses such as appetite regulation, mood, intestinal inflammation. Vagal stimulation is already an established treatment for resistant mood dosorders and is being explored for potential substance use disorder-related behavioral modification (Altschuler et al. 1993; Berthoud et al. 1991; Bremner 2020; Childs et al. 2017; Matteoli and Boeckxstaens 2013).

Several studies have explored microbiome signaling through vagal afferents by observing changes in the immediate early gene cFOS, which labels active neurons in the vagal ganglia and the brain. Activation of neurons has been observed using this technique following oral administration of various microbes (Goehler et al. 2005; Bharwani et al. 2020). Functional studies have demonstrated that oral exposure of *Lactobacillus Rhamnosus* decreases anxiety-like behavior in mice, which is abolished by vagotomy (Cawthon and Serre 2018). In contrast, activation of the vagus nerve increases in the production of indole, a proinflammatory microbial metabolite that increases anxiety-like behavior (Jaglin et al. 2018). However, stimulation parameters and behavioral paradigms can result in differential behavioral outcomes. Childs et al. used vagus nerve stimulation to extinguish appetitive behaviors and reduced relapse in a model of cue-induced cocaine self-administration reinstatement in rats (Childs et al. 2017). Intermittent blockage of vagus nerve signaling is being explored as an effective form of weight loss by short term control of eating (Pelot and Grill 2018). Furthermore, regions of the brain associated with eating behavior linked with obesity are the same regions associated with substance use disorder-related behaviors. Both share similar neuronal substrates in natural reward centers and disrupted signaling results in loss of control and disorder progression. Further research is needed, but these observations suggest that vagal signaling has both a direct and indirect role in modulating the gut-brain axis and downstream behaviors.

## The Immune System and the Microbiome

The microbiome is linked to the immune system from the beginning of life. Our earliest commensal microbes educate the immune system as we develop, act as a shield against pathogenic bacteria, and directly signal to immune related cells in the gut (Francino 2014). Therefore, it is likely that drug induced changes to microbiota can influence immune response and vice versa. There are various mechanisms by which the microbiota and its products are linked to the immune system, including vagal signaling (Bonaz et al. 2017; Goehler et al. 1999), microbial epitopes, and metabolite production. Innate immune cells that come into contact with the microbiota express membrane and intracellular proteins that sense microbial molecules. Microbe associated molecular patterns (MAMPs) such as LPS, lipoteichoic acid (LTA), and peptidoglycans induce proinflammatory cytokines (e.g., IL-1β, IL-6, TNF-a) by activation of Toll-like receptors (TLRs) and Nod-like receptors (NLRs) (Thaiss et al. 2016).

Interferon-1 (IFN-1) modulation occurs through microbial metabolites such as acetate, a short-chain fatty acid (SCFA) which is primarily produced through the digestion of dietary fiber by the microbiome. The microbiome also modifies neutrophil function and can impact the differentiation of T-cells into a variety of T-helper cells (Francino 2014). Additionally, not only is immunological receptor availability modified by the microbiota, but also the overexpression or lack of receptors can result in changes to the microbiome itself. TLRs that are typically associated with targeting a specific microbes via MAMPs can confer a benefit to the surrounding microbial community by supporting or limiting that microbe, or conversely this change can potentially initiate dysbiosis through the same pathways (Vijay-Kumar 2010). The microbiome-immune axis has been explored in relation to interactions in affective behaviors (Sylvia and Demas 2018); however, a broader discussion of the microbiome-immune axis beyond the scope of this review. There is a need for an in-depth examination of the connection between the brain, gut, and immune system and its role in physiological conditions such as addiction. This review will focus on the specific alterations of the immune system for each drug of abuse and how increased inflammation, degradation of the gut lining, and dysbiosis exacerbate drug taking behavior. For further review on the microbiome-immune axis see the following publications (Thaiss et al. 2016; El Aidy et al. 2014; Maranduba et al. 2015; Fung 2020; Salvo-Romero et al. 2020). Figure 1 provides a broad overview of the microbiome and related immunological, metabolite signaling, and organ function in healthy and dysbiosis states.

## Functional Signaling Metabolites Produced by the Microbiome

Metabolite production, including that of short-chain fatty acids, bile acids, and neurotransmitters, are another mechanism by which the gut-brain axis can modify substance use disorders (Cryan and Dinan 2012). Aside from producing bioactive molecules, microbes have been shown to metabolize drugs and functionalize/deconjugate circulating metabolites (Swanson 2015; Zimmermann et al. 2019). These mechanisms can modify the drug's effectiveness and pharmacokinetics, which can alter the valence of reward and withdrawal symptoms. Secondary functionalization of metabolites may also increase the prevalence of toxic byproducts that contribute to liver toxicity, intestinal permeability, and inflammatory responses, all of which can contribute to downstream behavioral changes and disease severity (Wilson and Nicholson 2017; Caldwell and Hawksworth 1973; Clarke et al. 2019). While the impact of microbial metabolism on prescription drugs is currently applied to the development of pharmaceuticals (Hitchings and Kelly 2019), it may also be relevant to the field of substance use disorders to understand better potential avenues by which microbes may impact substance use disorders.

### Short-Chain Fatty Acids

SCFAs, one of the principal families of microbial metabolites, result from the fermentation of dietary fiber in the gut by the resident microbiota and can influence brain function and immune responses (Silva et al. 2020; Dalile et al. 2019). As signaling molecules and energy sources, SCFAs modulate tight junctions in the gut epithelium, leukocyte development, and regulate several leukocyte functions, including the production of cytokines, chemokines, and eicosanoids (Correa-Oliveira et al. 2016; Vinolo et al. 2011a). Behaviorally, administration of SCFAs in preclinical models of stress has been found to alleviate stress-responsiveness, reduce anhedonia, and stress-induced intestinal permeability (Van De Wouw 2018). SCFAs further function as ligands of G protein-coupled receptors (GPCRs) FFAR2, FFAR3, GPR109, and Olfr78 (Kimura et al. 2011; Priyadarshini et al. 2018) and also as histone deacetylase inhibitors, which significantly impact behavior and gene transcription (Silva et al. 2020; Correa-Oliveira et al. 2016; Bourassa et al. 2016; Yuille et al. 2018; Licciardi et al. 2011). These functions have been extensively studied and are well-reviewed (Vinolo et al. 2011a; Antunes et al. 2019; Ohata et al. 2005; Parada Venegas et al. 2019).

### Bile Acids

Bile Acids are steroid acids produced by the liver. These compounds act as a significant regulator of the microbiome through direct amphipathic antimicrobial action on microbial membranes (Begley et al. 2005), and indirectly via Farnesoid receptor (FXR)-induced peptides (Inagaki et al. 2006). Bile acids are crucial signaling molecules that influence immune homeostasis, with overexpression of certain bile acids causing inflammation and even cell death (Chiang 2013). Microbes directly regulate the available pool of bile acids by deconjugating primary bile acids into secondary bile acids. Secondary bile acids are essential for the emulsification of fats for later absorption in the intestine, altering satiety and energy expenditure profiles (Ramirez-Perez et al. 2017; Wu et al. 2020). Conditions of dysbiosis can cause a shift in the production of secondary bile acids, resulting in an over-abundance of primary bile acids (Staley et al. 2017). Bile acid concentrations also preferentially alter the makeup of the microbiome causing degradation of gram-positive cell walls that lack the outer layer of protection of LPS. High concentrations of bile acids can also be detrimental to the host, causing oxidative stress, DNA damage, and cell death resulting in intestinal permeability (Payne et al. 2008). An increase in intestinal permeability increases peripheral inflammation and pathways that lead to further dysbiosis. For substance use disorders such as alcohol, a degradation of proper liver functioning can drastically alter alcohol metabolism, bile acid availability, and disease progression severity. For further review of the function and impact of bile acids on the microbiome (see Ridlon et al. 2014).

### Neurotransmitters

Neurotransmitters such as dopamine, serotonin, epinephrine, norepinephrine, gamma-aminobutyric acid (GABA), and acetylcholine play an essential role in the signaling and homeostasis of the body (Mittal et al. 2017). From gut motility to nutrient absorption, many neurotransmitters are produced and used in the gut by microbes (Strandwitz 2018) and gastrointestinal related-cells (Cooke 2000). Dysregulation of neurotransmitters in the gut (particularly serotonin) is a common drug-related side effect. Peripheral neurotransmitter dysregulation is also linked other disease states such as inflammatory bowel disease and Parkinson’s disease (Ghia et al. 2009; Kidd et al. 2009; Dinan and Cryan 2017). Similar to peripheral serotonin production, the microbiome also serves as an alternative route for kynurenic acid production, an endogenous tryptophan metabolite Dehhaghi et al. (2019). In the periphery, kynurenic acid excites dorsal root ganglia neurons through the activation of GPR35 (Cosi et al. 2011), impacting the perception of pain. Kynurenic acid can also reduce TNF⍺ expression, secretion and downstream immune signaling. In the central nervous system, kynurenic acid acts as a neuromodulator, interacting with M-Methyl-D-aspartic acid (NMDA) (Kessler et al. 1989) and nicotinic receptors (Hilmas et al. 2001), modulating the release of other neurotransmitters such as glutamate (Carpenedo et al. 2001), acetylcholine (Albuquerque and Schwarcz 2013), and dopamine (Ramos-Chavez et al. 2018; Okuno et al. 2011). For additional review of kyurenic metabolism and its physiological impacts, see Wirthgen et al. (Wirthgen et al. 2017). Other critical signaling molecules to consider are neuropeptides, including neuropeptide Y, substance P, corticotrophin-releasing factor, and vasoactive intestinal polypeptide (among many others) (Holzer and Farzi 2014). Apart from acting as neurotransmitters, neuropeptides also commonly function as gut-hormones through GPCRs. Gut hormone signaling does not always occur through endocrine pathways but also from activation of the vagus nerve (Holzer and Farzi 2014). There is still much to be explored surrounding the interconnected nature of microbial metabolites in the modulation of the processes that impact behavior, with the potential to modify the microbiome or leverage metabolites to have clinically beneficial outcomes.

## Potential Clinical Applications of the Gut-Brain Axis

Alterations of the gut microbiome have been implicated in autism spectrum disorder (Vuong and Hsiao 2017), major depressive disorder (Kelly et al. 2016; Kelly et al. 2019), Alzheimer’s disease (Kowalski and Mulak 2019) and addiction (Meckel and Kiraly 2019; Ren and Lotfipour 2020; Wang et al. 2020) among others. Therefore there is considerable interest in leveraging the gut microbiome to support human health and mental well-being. Studies have also linked the microbiome to several fundamental neurological underpinnings, including synaptic plasticity (Leung and Thuret 2015), neuroinflammation Cerovic et al. (2019), and neurotransmitter signaling. Conversely, patients with gut-related disorders such as inflammatory bowel disease experience dysregulation of sleep, have a high incidence of depression, and increased rates of anxiety (Limbana et al. 2020; Bannaga and Selinger 2015). To address this issue, everything from the use of pro- and pre-biotics to fecal transplantation and antibiotic therapies is being explored to treat CNS-related diseases.

Fecal microbiome transplants (FMTs) are employed to replicate an intact sampling of the microbiome rather than supplementation with a single species or small group of microbes. However, there are significant caveats to fecal transplants. The microbiome includes fungi, viruses, and phages, making it difficult to determine how the whole community may impact the donor and match donors with patients (Sbahi and Palma 2016). Live biotherapeutics (LBP) are a biological product that contains live organisms for the treatment of disease, this can be a probiotic with naturally occurring strains, or a modified organism. LBPs are gaining traction and efforts are being made to regulate and develop them (Cordaillat-Simmons et al. 2020). However, in both cases of FMT and LBPs, it can be a challenge for newly supplemented microbes to find a niche due to extreme competition in the gut.

Diet-induced or antibiotic-induced modulation of the microbiome is much easier to achieve in the laboratory setting. Outside of the laboratory pro- and pre-biotics are widely used because dietary changes are often difficult to maintain. Administration of probiotics such as *Lactobacillus* and *Bifidobacterium* have emerged as promising treatments to reduce gut leakiness (Rao and Samak 2013), endotoxin levels (Wang et al. 2006), and attenuate the hypothalamic-pituitary axis (HPA) response to stress through the modulation of biologically active molecules such as serotonin (Sarkar et al. 2016), norepinephrine (Cao et al. 2018), brain-derived neurotrophic factor (BDNF) (Liang et al. 2015), cortisol (Liang et al. 2015), all of which are involved in potentiating addiction relapse events. The quantity of data supporting microbiome manipulation of behavior showcases the potential for analogous microbiota interventions in the context of addiction and related behavioral tendencies. To further explore mechanisms and potential connections between the microbiome and substance use disorders, the remainder of this review will focus on the literature surrounding specific drugs of abuse and their related microbiome impact. For ease, a comprehensive table of microbiome manipulations that are related to behavior is included in Table 1.

**Table 1:** **Microbiome manipulations and substance use disorders.** This table outlines the current literature of direct microbial manipulations and SUD related behaviors in preclinical animal models

Species	Drug	Administration	Manipulation	Behavioral Effect	Citation
Mouse	Cocaine	Passive (IP)	Antibiotics SCFA + Antibiotics	↑ Locomotor sensitization ↑ CPP SCFA administration Rescues behavior changes	(Kiraly et al. 2016)
Mouse	Cocaine	Passive (IP)	Surgical intervention to increase bile acid	↓ Locomotor Sensitization ↓ CPP	(Reddy et al. 2018)
Mouse	Cocaine Morphine	Passive (IP) Passive (Pellet)	Antibiotics	↓ CPP ↓ Latency in tail immersion test	(Lee et al. 2018a)
Mouse	Morphine	Passive (Pellet)	Antibiotics	Prevented antinociceptive tolerance in tail immersion test and acetic acid stretch assay	(Kang et al. 2017)
Mouse	Morphine	Passive (injections twice daily)	DHA supplementation	↓ Anxiety (EPM) - Thermal Analgesia	(Hakimian et al. 2017)
Rat	Oxycodone	Passive (IM)	Antibiotics	Altered Neuronal Ensembles recruited during intoxication and withdrawal	(Simpson et al. 2020)

## Alcohol and the Microbiome

Alcohol has been a part of human culture for over a millennium; however, excessive alcohol consumption is one of the leading causes of mortality worldwide, accounting for nearly 6% of total deaths (GBD 2016 Alcohol Collaborators 2018; Rehm and Shield 2019; Bardach et al. 2019). AUD results from a complex interaction of social, environmental, and genetic factors and can lead to long-term negative outcomes to the central nervous system and peripheral organs. Chronic alcohol intake leads to small and large intestinal bacterial overgrowth, and community shifts in the oral and gut microbiome which results in dysbiosis that has been observed in humans and preclinical animal models (Bode et al. 1984; Casafont et al. 1996; Yan et al. 2011; Hartmann et al. 2013; Engen et al. 2015; Bull-Otterson et al. 2013; Leclercq et al. 2014; Yussof et al. 2020).

Alterations of the microbiome are thought to be a critical pathway for the development and maintenance of alcohol use disorder and associated alcohol liver disease (ALD) (Tripathi et al. 2018; Dubinkina et al. 2017). AUD and ALD progression are correlated to increased intestinal permeability (Leclercq et al. 2014), altered production of bile-acids (Bajaj and Hylemon 2018), other metabolites/signaling molecules (Zhong and Zhou 2014), genetic factors (Anstee et al. 2015; Meroni et al. 2018; Stickel et al. 2017), and changes in circadian rhythm (Bajaj et al. 2017). Importantly, dysbiosis is associated with the progression of cirrhosis of the liver (Chen et al. 2011), worsening of comorbid psychiatric disorders (Petra et al. 2015), and nutritional deficiencies Hibberd et al. (2017). Not only is the gut–brain axis altered in patients with AUD, but it can also negatively influence disorders often found to be comorbid with substance abuse, such as eating and anxiety disorders (Temko et al. 2017; Xiao et al. 2018; Volpe et al. 2014).

### Alcohol Use and Microbes

High levels of alcohol consumption are linked to shifts in the microbiome, available amino acids (Tedesco et al. 2018), and increased inflammation (Kakiyama et al. 2014). Though, method of intake and type of alcohol directly impact the outcome of microbial modifications. Mice fed alcohol (ethanol) via intragastric administration exhibit a reduction of beneficial bacteria from the phyla Firmicutes and an expansion of Verrucomicrobia and Bacteroidetes compared to controls (Yan et al. 2011). Similarly, rats that voluntarily consumed alcohol exhibit reduced alpha diversity (Kosnicki et al. 2019). In contrast, mice fed fermented rice liquor exhibit an expansion of *Turicibacter,* which is known to bidirectionally communicate with the host serotonergic system (Fung et al. 2019). Interestingly, animals exposed to fermented rice liquor exhibited increased SCFA production, likely due to the fermented nature of the beverage (Lee et al. 2020). In this instance, the method of fermentation of the beverage may outweigh some of the adverse effects of drinking on SCFA production.

In the clinic, alcoholics with cirrhosis exhibit reductions in Bacteroidetes and increases in pro-inflammatory Proteobacteria and Fusobacteria (Chen et al. 2011). Alcoholics with mild liver disease exhibited similar reductions of *Lactobacillus* spp. and *Bifidobacterium* spp. (Leclercq et al. 2014). Not all changes were permanent, however; individuals that followed a 19-day abstinence period demonstrated a rebound of some microbes, including *Ruminococcaceae* (Leclercq et al. 2014). While some degree of alcohol-induced dysbiosis is potentially long-lasting, the resulting effects may also hinge on the individual’s baseline microbiome composition. Overall, a decrease in SCFA producing microbes and increases in proinflammatory microbes were observed in both human and animal models of AUD. Supplementation of SCFAs or other yet unidentified metabolites may be leveraged to reduce adverse outcomes in AUD. Table 2 includes an inclusive list of drug-related perturbations to microbes, metabolites, and immune-related markers.

**Table 2:** Impact of Drugs of Abuse on Microbiome, Metabolite, and Immune Markers. This table outlines the current literature on the effects of alcohol, opioids, stimulants, psychedelics, THC, and CBD on the microbiome

Species	Sample Site	Drug	ROA	Δ Microbiome	Δ Metabolites	Δ Immune response	Citation
Rat	Intestine Liver	Alcohol	Oral	Testing of LGG supplement	-	LGG supplement reduced liver health and IP to near control levels ↑ Oxidative stress ↑ Carbonyl ↑ Nitrotyrosine	(Forsyth et al. 2009)
Mouse	Small Intestine Large Intestine	Alcohol	Oral	*↓ Lactobacillus* *↓ Lactococcus* *↓ Leuconostoc* *↓ Pediococcus* *↑ Bacteroidetes* *↑ Verrucomicrobia* *↑ Akkermansia*	-	↓ Reg3b ↓ Reg3g ↓ Defensin 5 ⍺	(Yan et al. 2011)
Mouse	Feces	Alcohol	Oral	*↓ Firmicutes* *↓ Bacteriodetes* *↑ Proteobacteria* *↑ Actinobacteria* (*4)	↑ Endotoxins	Endotoxemia reduced by Lactobacillus rhamnosus (LGG) probiotic ↑ TNF-alpha	(Bull-Otterson et al. 2013)
Mouse	Feces	Alcohol	Oral	↓ *Lactobacillus* ↑ *Akkermansia Muciniphilia*	-	-	(Hartmann et al. 2013)
Mouse	Feces	Alcohol	Intragastric	↓ *Lactobacillus*	↓ LCFA	↓ Genes involved in the biosynthesis of saturated fatty	(Chen et al. 2014)
Mouse	Feces	Alcohol	CIE	*↑ Alistipes* *↓ Clostridium IV* *↓ Clostridium XIVb* *↓ Coprococcus* *↓ Dorea* *↓ Alpha Diversity (CIE)* *↑ Akkermansia* (CIE)	Decrease in SCFA producers	Similar reductions in *Dorea* and *Coprococcus* in chronic social stress models have been correlated to increases in proinflammatory cytokines IL-6 and MCP-1 (Bailey et al. 2011)	(Peterson et al. 2017)
Mouse	Feces Colon	Alcohol	Oral	*↓ Bacteroidetes* *↓ Firmicutes* *↑ Proteobacteria*	-	↑ IL-1β	(Kang et al. 2017)
Rat	Jejunum Colon	Alcohol	Oral	*↑ Bacteroidetes* *↓ Firmicutes* *↑ Proteobacteria*	↑ Blood Endotoxin	↑ Amino Acid metabolism (arginine and proline)	(Fan et al. 2018)
Mouse	Feces Liver Colon	Alcohol	Oral	*↑ Bacteroidetes* *↑ Verrucomicrobia* *↑ Firmicutes* *↓ Ruminococcaceae* *↑ Odoribacter*	No significant change in SCFA	↑ Serotonin ↑ Taurine ↑ Bile acid level	(Wang et al. 2018b)
Rat	Feces	Alcohol	Oral	↓ Diversity *↓ Lactobacillus* *↓ Peptostreptococcaceae* *↓ Turicibacter* *↑ Parabacteroides* *↑ Barnesiellaceae* *↑ Bacteroides*	-	-	(Kosnicki et al. 2019)
Mouse	Feces	Alcohol	Intragastric	*↓ Bacteroidetes* *↑ Firmicutes* *↓Muribaculum intestinales*	-	-	(Lee et al. 2020)
Human	Jejunum	Alcohol	Oral				(Bode et al. 1984)
Human	Feces	Alcohol	Oral	*↓ Bifidobacteria* *↓ Enterococci* *↓ Lactobacilli*	-	-	(Kirpich et al. 2008)
Human	Feces	Alcohol*(1)	See below	*↓ Bacteroidetes* *↑ Proteobacteria* *↑ Fusobacteria*	-	-	(Chen et al. 2011)
Human	Colon	Alcohol	Oral	*↓ Bacteroidetes* *↑ Proteobacteria*	↑ Endotoxin	↑ Cytokines ↑ Oxidase	(Mutlu et al. 2012)
Human	Urine	Alcohol	Oral	-	↑ Blood LPS	↑ TNF⍺ ↑ IL-6	(Leclercq et al. 2012)
Human	Feces, Urine	Alcohol	Oral	*↓ Lactobacillus* *↓ Bifidobacterium* (reversed during withdrawal) *↓ Ruminococcae* (*5) *↑ Lachnospiraceae* (*5) *↓ F. Prausnitzii* (*5)	MCFA lower in control and withdrawal, Phenol higher in AUD (*2)	Drawing connection of leaky gut (IP) to dysbiosis	(Leclercq et al. 2014)
Human	Feces	Alcohol	Oral	↑ *Bacteroidetes* *↓ Firmicutes*	-	-	(Volpe et al. 2014)
Human	Feces	Alcohol	Oral	-	↑ Tetradecane ↓ Fatty alcohols ↓ Propionate ↓ Isobutyrate ↓ Caryophyllene ↓ Camphene ↓ Dimethyl-disulfide ↓ Dimethyl-trisulfide	-	(Couch et al. 2015)
Human	Feces	Alcohol	Oral	*↑ Proteobacteria* *↓ Faecalibacterium* *↑ Sutterella* *↑ Holdemania* *↑ Clostridium*	↓ Butyric acid	-	(Bjorkhaug et al. 2019)
Mouse	Feces	Cocaine	IP	*↓ Mucispirillum* *↓ Ruminococcaceae* *↓ Lachnospiracea* *↓ Pseudoflavonifractor* *↓ Butrycicoccus*	-	↑ NF-κB ↑ IL-1β ↑ IL-18 ↑ CCL-2 ↑ CCL-7 ↑ CXCL-10 ↑CCL-11	(Chivero, et al. 2019)
Rat	Feces	Cocaine	Volatized	↓ Alpha Diversity ↓ Beta Diversity	↓ Aromatic amino acid decarboxylase gene	-	(Scorza et al. 2019)
Human	Feces	Cocaine	Active Users	↑ *Bacteroidetes* *↓ Firmicutes*	No Change in Blood LPS	↑ Interferon-γ	(Volpe et al. 2014)
Rat	Feces	Methamphetamine	IP	↑ Diversity *↓ Acidaminococcaceae* *↓ Phascolarctobacterium* *↑ Ruminococcaceae*	↓ Propionate	-	(Ning et al. 2017)
Human	Rectal swab	Methamphetamine	Active users	*↑ Finegoldia* *↑ Parvimonas* *↑ Peptoniphilus* *↑ Porphyromonas* *↓ Butyricicoccus* *↓ Faecalibacterium*	-	-	(Cook et al. 2019)
Human	Feces	Heroin Methamphetamine Ephedrine	Active Users	*↑ Thauera,* *↑ Paracoccus* *↑ Prevotella*	Not examined	-	(Xu et al. 2017)
Human	Feces	Opioids	Active Users	↑ Alpha Diversity	-	-	(Vincent et al. 2016)
Human	Feces	Opioids	Active Users	*↓Bacteriodacea* *↓Clostridiales XI* *↓Ruminococcaceae*	↑ Amino Acid metabolism ↑ Degradation of BCAA	↑Endotoxemia ↑ IL-6	(Acharya et al. 2017)
Human	Feces	Opioids	Active Users	*↑ Bifidobacterium* *↑ Prevotella*	-	-	(Barengolts et al. 2018)
Mouse	Blood Lavage	Morphine	Passive Exposure model of sepsis	↑*Staphylococcus* ↑*Enterococcus*		↑IL-17 vial TLR2 ↑Intestinal inflammation	(Meng et al. 2015)
Mouse	Feces	Morphine	Passive Exposure	↓ *Bacteroidetes* *↑ Firmicutes*	↓ Primary Bile Acids ↓ Secondary Bile Acids	↑IL-17 ↓IL-10	(Banerjee et al. 2016)
Mouse	Feces	Morphine	Passive Exposure	↑ *Enterococcus Faecalis*	↓ Bile Acids (DCA) ↑ Saturated Fats ↑ Phospytidylethanolamine (PE)	-	(Wang et al. 2018a)
Mouse	Feces	Morphine	IP (intermittant and sustained)	*↑ Ruminococcus (int)* *↓ Lactobacillus (int)* *↑ Clostridium (sust.)* *↑ Rikenellaceae (sust.)*	-	-	(Lee et al. 2018a)
Mouse	Feces	Oxycodone	IVSA				(Hakimian et al. 2019)
Non Human Primate	Feces	Morphine	Passive	*↑ Methanobacteriaceae* *↓ Streptococcaceae* *↓ Pasturellaceae*	↓ Primary Bile Acids ↑ Secondary Bile Acids	-	(Sindberg et al. 2019)
Rats	Caecum	Nicotine	Oral	↓ *Bifidobacterium*	↓ Acetic acid ↓ Propionic acid ↓ Butyric acid ↓ Valeric acid	-	(Tomoda et al. 2011)
Mice	Large intestine	Nicotine	Oral	↑ *Clostridium clostridiforme* ↓ *Lactoccoci* ↓ *Ruminococcus* ↓ *Enterobacteriaceace* (Relative abundances)	-	↓ Intestinal inflammatory response ↓ Activation of nuclear factor-κβ	(Wang et al. 2012)
Mice	Colon, ileum	Nicotine	Oral	↑ *Lachnospiraeae* (colon)	-	↑ Cxcl2 (ileum) ↓ IFN-γ (ileum) ↑ IL-6 (colon) ↓ TGF-β (colon)	(Allais et al. 2016)
Human	Feces	Nicotine	Oral	↑ *Prevotella* ↑ *Bacteroides*	-	-	(Benjamin et al. 2012)
Human	Feces	Nicotine	Oral	↓ *Firmicutes* ↑ *Bacteroidete*s ↑ *Proteobacteria* ↓ *Actinobacteria*	-	-	(Biedermann et al. 2013)
Human	Upper intestinal tract	Nicotine	Oral	↑ Alpha diversity ↑ Beta diversity ↑ *D. invisus* ↑ *M. micronuciforms*	-	-	(Vogtmann et al. 2015)
Human	Mouth	Nicotine	Oral	*↓ Proteobacteria* *↓ Capnocytophaga* *↓ Peptostreptococcus* *↓ Leptotrichia* *↑ Atopobium* *↑ Streptococcus*	-	-	(Wu et al. 2016)
Human	Feces	Nicotine	Oral Gut	↓ Shannon diversity (Fecal) ↑ *Prevotella* ↓ *Bacteroides*	-	-	(Stewart et al. 2018)
Human	Feces	Nicotine	Oral	*↑ Bacteroidetes* *↓ Firmicutes* *↓ Proteobacteria*	-	-	(Lee et al. 2018b)
Human	Upper intestine	Nicotine	Oral	↑ *Firmicutes* ↑ *Rothia* ↓ *Prevotella* ↓ *Neisseria* ↓ Diversity (relative abundance)	-	-	(Shanahan et al. 2018)
Human	Ileum	None (testing Chron’s disease (CD))	NA	↓ *F.Prausnitzii* associated with higher CD		↓ IL-12 ↓ IFNɣ ↑ IL-10 Colitis Dysbiosis mediated with *F. Prausnitzii* supplement	(Sokol et al. 2008)
Mouse	Feces	Cannabis	Oral	↑ *Akkermansia* ↑ *Firmicutes:Bacterioides*		THC blocked weight gain from high fat diet	(Cluny et al. 2015)
Human	-	Cannabis	-	-	-	Highly palatable food increased endogenous cannabinoids was associated with hedonic eating	(Monteleonei et al. 2012)
Rat	-	Cannabis	-	-	-	Fasting ↑ anandamide	(Gomez et al. 2002)

### Alcohol Use and Alterations of Microbial Metabolites

Separating the changes in metabolism and metabolites that are related to alcohol intake versus microbiome variability can be challenging. For example, amino acids such as threonine and glutamine and bile acids such as guanidinosuccinate and isocitric acid are elevated in plasma as a result of alcohol metabolism rather than microbial metabolism (Harada et al. 2016; Rachakonda et al. 2014). Comorbid diseases also contribute to complex signaling. Patients with alcoholic hepatitis exhibit increases in levels of metabolites related to lipolysis and oxidative stress in serum due to decreased liver function (Rachakonda et al. 2014). In contrast, a reduction in microbiota-associated bile acids and an increased concentration of conjugated primary bile acids are observed in alcoholics as a result of microbial metabolism (Ridlon et al. 2014). Shifts in available bile acids result in alterations of gram-positive bacterial species that are more sensitive to bile acid production due to cell wall composition. Bile acid imbalance is detrimental to the larger microbial community as many gram-positive species are also producers of SCFAs that downregulate proinflammatory signals, strengthen tight junctions, and inhibit colonization by pathogenic microbes. The prophylactic administration of SCFAs has been shown to mitigate chronic-binge ethanol-induced intestinal barrier and liver injury (Cresci et al. 2017). Unsurprisingly, Gut-microbiota-associated metabolites vary based on level of alcohol use (Leclercq et al. 2017).

Couch et al. (2015) hypothesized that intestinal microbiota function might be altered in alcoholics, leading to increased alcohol-associated pathologies. They examined metabolites in the feces of alcoholics and found decreased SCFAs in alcoholics versus non-alcoholic controls. Alcoholics exhibit decreased SCFA production and the loss of butyrogenic bacteria. This shift in community makeup is also found in inflammatory diseases like psoriatic arthritis and inflammatory bowel disease (Scher et al. 2015; Wang et al. 2014). Broadly, SCFAs producing bacteria and SCFA production are reduced in conditions of dysbiosis (Lloyd-Price et al. 2019). Bajaj et al. administered FMTs from a donor enriched for SCFA producing *Lachnospiraceae* and *Ruminococcaceae* to patients with AUD related cirrhosis and problem drinking and observed that craving was reduced by 90% in the FMT group versus 30% in the control. There was a reduction in serum IL-6, and LPS, as well as increases in SCFAs in the treated group (Bajaj et al. 2020). These observations underscore that not only are metabolite shifts in response to alcohol are potentially harmful to the makeup of the resident microbial communities, but also that microbial metabolites are merely the top of a cascade that broadly impacts other pathways which magnify complications of dysbiosis, such as the activation of immune responses within the gut.

### Alcohol Use and the Immune System

Clinicians have long acknowledged an association between excessive alcohol intake and immune-related health outcomes. Alcoholics have a greater likelihood of experiencing liver disease, cancer, slow wound healing, and sepsis (Sarkar et al. 2015). However, a caveat of comparing the human and rodent immune responses is that there are many differences between them. Rodents express more TLR’s than humans, and there are species-specific differences in LPS stimulation (Rehli 2002). As long as the models' limitations are taken into consideration, they are still useful for exploring the role of the gut-brain axis in AUD in a controlled setting that is often unachievable in humans. Multu et al. (2012) hypothesized that chronic alcohol consumption would result in alterations of the gut microbiome and that these changes may be responsible for increased inflammation and endotoxemia. Indeed, individuals that exhibited lower concentration of SFCA producing Bacteroidetes, and high concentration of inflammation inducing Proteobacteria appear to be highly correlated with the onset of endotoxemia. Of all drugs of abuse discussed in this review, increases in Proteobacteria is seen in patients with substance use disorders in four out the of six discussed substances.

A healthy mucosa and an intestinal layer is essential to controlling the translocation of negative signals from the microbiome to the rest of the body. A lack of intestinal permeability and diverse commensal communities also restrain the expansion of pathogenic bacteria (Turner 2009). Intestinal permeability allows for microbial antigens to circulate widely which increases inflammatory cascades. This indicates that changes to the microbiome in AUD directly affect the immune function. Increases endotoxin-producing Proteobacteria bacteria such as Enterobacteriaceae and a decrease in SCFA producing Firmicutes such as *Ruminococcaceae* and *Lachnospiracea* (Bull-Otterson et al. 2013; Wang et al. 2011; Chen et al. 2015; Forsyth et al. 2009) are associated with increased alcohol intake. An increase in proinflammatory microbes contributes to further degradation of the intestinal barrier and alteration of the commensal bacteria that comprise the defense system of the gut. Similarly, Yan et al. demonstrated that alcohol feeding in mice led to microbial overgrowth and dysbiosis with increases in Bacteroidetes and Verrucomicrobia and a decrease in Firmicutes (Yan et al. 2011). Interestingly an overgrowth of *Akkermansia Muciniphila* was observed in this model and is hypothesized to play a role in the degradations of mucins that can lead to intestinal permeability, despite its typical association as a beneficial microbe (Yan et al. 2011).

### Alcohol Use and Intestinal Permeability

In humans, Leclerq et al. found that alcoholics can be divided into two groups: high and low intestinal permeability. While both AUD groups showed a higher prevalence of psychiatric disorders such as depression and anxiety, rates were higher with patients exhibiting increased intestinal permeability (Leclercq et al. 2014). Patients without dysbiosis did not exhibit similar permeability despite heavy alcohol consumption (Leclercq et al. 2014). Dependent subjects with intestinal permeability also showed a decreased abundance of *Ruminococcus, Fecalibacterium prausnitzii, Oscillibacter*, and *Anaerofilum.* In particular, *Fecalibacterium prausnitzii,* which is known to be correlated with reduced inflammatory response, was significantly decreased in individuals with increased intestinal permeability, and levels did not change at the end of the abstinence period. These individuals also exhibited an increase in other *Lachnospiraceae*, *Blautia, and Megasphaera* which have been associated with hepatic encephalopathy and impaired cognition (Leclercq et al. 2014; Dhiman 2012).

Preclinical models have employed germ-free mice that received fecal transplantation from human alcohol-dependent subjects with severe alcoholic hepatitis (sAH) or no alcoholic hepatitis (nAH) to study how the gut microbiota play a role in the modulation of intestinal permeability and the development of alcohol-induced liver disease (Llopis et al. 2016). Increases in *Bifidobacteria* and *Streptococci* with differential expression of many members from the *Lachnospiraceae* family were observed in animals from the sAH group, compared to the nAH group. The nAH group exhibited increased levels of *Akkermansia, Turcibacter*, and *Phascolarctobacterium*, as well as members of the *Ruminococcaceae* family, which are known for anti-inflammatory properties and maintaining a health mucosal layer (Sokol et al. 2008; Forbes et al. 2016*).* Likely, these microbes conferred a reduction in bacterial epitope translocation and downstream immune system activation, as well as protection from alcohol-induced hepatitis.

There are conflicting reports as to whether AUD-induced intestinal permeability is long-lasting, which is relevant to potential microbiome interventions. Several studies have shown that AUD-mediated increases in intestinal permeability reverse following alcohol abstinence (Maccioni et al. 2020; Ohlsson et al. 2019; Flux and Lowry 2020; Peirce and Alvina 2019). Alcohol-associated intestinal permeability was also reversed in rodent models through the application of probiotics (*Lactobacillus Rhamnosus*) (Forsyth et al. 2009) and the administration of microbial metabolites such as SCFAs. Increased intestinal permeability can allow for LPS to cross the gut epithelium and activate downstream immunological pathways. Monocytes from humans with ALD also exhibit priming for the release of cytokines (Hunt and Goldin 1992). An increase of TNF-alpha, IL-6, and IL-8 have also been documented in AUD patients (Leclercq et al. 2014). This topic has been extensively reviewed by Leclerq et al. and others (Leclercq et al. 2017).

Altogether, alcohol use has a significant impact on the microbiome and downstream inflammatory pathways involved in liver and comorbid CNS disease severity. Preclinical supplementation of microbes and microbial metabolites such as SCFAs suggests that microbiome-related treatments could provide several avenues to develop novel therapies for AUD and liver-related comorbidities. While there may not be one AUD-related microbiome, further exploration of the gut-brain axis and AUD may contribute to harm reduction and improve behavioral outcomes.

## Opioids and the Microbiome

Despite being an effective analgesic, the rewarding and euphoric effects of opioids reinforce early use and the negative emotional and physical outcomes related to withdrawal considerably increase abuse liability (Koob 2020). These properties have led to a public health crisis with high rates of relapse (Smyth et al. 2010) and approximately 150 opioid-overdose related deaths per day CDC/NHS (2020). Many treatments have been explored to reduce the negative outcomes related to opioids, however few studies have explored the role of the microbiome in opioid use. Some of the first points of contact for opioids are in the gut. Opioid receptors are widely expressed throughout the gastrointestinal tract on neurons within the myenteric and submucosal plexus (Galligan and Akbarali 2014). Activation of µ-receptors (mu) by opioids reduces gut motility and leads to opioid-induced constipation, one of the main complaints of those using opioids to manage pain (Camilleri et al. 2017; Argoff 2020). Clinically, studies have documented compositional shifts in the gut microbiota in individuals using opioids (Akbarali and Dewey 2019; Bell et al. 2009; Xu et al. 2017; Acharya et al. 2017). Dysbiosis induced by chronic opioid use is also linked to central opioid tolerance, acceleration of disease progression (Meng et al. 2015b), and immune modulation (Sacerdote 2006; Liang et al. 2016). Concurrent prescription of opioids and antibiotics or other prescription medicines may result in an additive effect on the microbiome and gut-brain communication Simpson et al. (2020). These preliminary observations support the need for more studies evaluating the role of the gut microbiome in opioid use disorder (OUD) progression and severity, as well as the potential use of the microbiome in the prediction and treatment of OUD.

### Opioid Use and Microbes

Preclinical models have identified that differences in gut microbiome composition are associated with route and schedule of opioid exposure. Rodents passively exposed to opioids via implanted morphine pellets exhibit elevated abundance of Firmicutes (Banerjee et al. 2016). Mice exposed to morphine passively demonstrated increases in gram-positive *Enterococcus faecalis,* a normal commensal that can cause sepsis and other infections if introduced to the bloodstream. Remarkably, blockade of the peripheral receptors attenuate community changes and resulting inflammatory pathways related to this microbe (Wang et al. 2018a). Intermittent morphine treatment significantly decreases the relative abundances of *Lactobacillus* spp. and increases *Ruminococcus* spp.; however, these taxa were unaffected following uninterrupted morphine treatment, suggesting that repeated opioid withdrawal bouts compound microbial changes in relation to OUD. In contrast, sustained but not intermittent morphine treatment increases the genus *Clostridium* (Lee et al. 2018a). Mice that receive chronic opioids exhibit an increase in proinflammatory genus S*taphylococcus*, *Enterococcus*, and Proteobacteria, and decreases in the abundance of beneficial genus Bacteriodales, Clostridiales, and Lactobacillales (Akbarali and Dewey 2019). Method of delivery, schedule of administration and model are involved in microbial shifts that are observed; however, an increase in inflammation and decrease in anti-inflammatory microbes appears to be a common result of opioid abuse as well as other drugs of abuse.

There are conflicting reports of opioid-related microbiome alterations in clinical populations. This discrepancy is likely due to heterogeneous samples, particularly related to polydrug use across samples. Barengolts et al. examined cross-sectional differences in opioid users compared to controls and reported a decrease in *Actinobacteria*, Bifidobacteriales, Lactobacillales, *Dialister*, and *Paraprevotella* with decreases in *Prevotella* and *Bifidobacterium* (Barengolts et al. 2018). Patients using opioid agonists exhibit a lower abundance of *Roseburia* (SCFA producer) and *Bilophila* (bile acid metabolizer), but there were no differences in patients using opioid antagonists. This finding parallels work in preclinical models that demonstrate drug use disrupts SCFA production and bile acid balance which are crucial reducing inflammation in the gut, and contribute to changes in drug-taking behavior (Gicquelais et al. 2020).

### Opioid Use and Metabolites

Systemic inflammatory factors originating in the gut might result in central nervous system effects through a compromised blood–brain barrier caused by chronic opioid use. Similar to shifts in *Roseburia* and *Bilophila* observed by Gicquelais et al. (2020) levels of SCFA’s are reduced by the peripheral μ-opioid receptor agonist and anti-diarrheal agent loperamide, perhaps due to a decrease in butyrate-producing bacteria (Touw et al. 2017). In the periphery, SCFAs act on GPCR’s free fatty acid receptors 2 (FFAR2) and 3 (FFAR3) to regulate leucocyte functions, such as the production of eicosanoids, chemokines, and cytokines involved in inflammatory responses (Vinolo et al. 2011b). In the CNS, opioid-induced microglia activation further leads to a reduction in the dopamine-dependent reward behavior via a BDNF signaling pathway (Taylor et al. 2016). Hakimian et al. hypothesized that withdrawal from opioids leads to microbiome depletion that can be rescued with supplementation of fatty acids, but instead of SCFAs, supplemented the long-chain polyunsaturated fatty acid (LCFA) docosahexaenoic acid (DHA) Hakimian et al. (2019). Supplementation of DHA blocked reinstatement of oxycodone self-administration in DHA-treated mice. Not only did the treatment impact behavior, treated animals exhibit increased richness and phylogenetic diversity following oxycodone exposure compared to untreated animals, which is generally accepted as beneficial. DHA administration also reduces anxiety-like behavior in mice following chronic morphine exposure, limiting a strong impetus for relapse behaviors (Hakimian 2017). Broadly, DHA supplementation has been explored for a variety of psychiatric illnesses and has been established to reduce anxiety-like behaviors, improve mood disorders, and other cognitive impairments in both rodent models (Pusceddu et al. 2015; Trofimiuk and Braszko 2013) and clinical settings (Ross et al. 2007; Zhang et al. 2016; Lesperance et al. 2011).

Another major driving factor for relapse in opioid models is increased pain during withdrawal. Several groups have demonstrated that metabolite pathways, including SCFAs, kynurenic acid, and bile acids, can alter pain-related processes (Csáti et al. 2015; Pineda-Farias et al. 2013; Mecs et al. 2009; Li et al. 2020). For instance, the administration of butyrate reduces nerve injury-induced pain (Guo et al. 2019) and oral administration of the SCFA butyrate was found to prevent morphine antinociceptive tolerance in mice treated with chronic morphine (Akbarali and Dewey 2019). The endogenous opioid system is crucial to sensation of pain, but also itch. Bile acids can modulate pain and itch by several mechanisms. TGR5, is a G-protein coupled bile acid receptor is be activated by bile acids to stimulate the release of endogenous opioids. The bile acids - deoxycholic acid, taurolithocholic acid, and oleanolic acid are all agonists of TGR5, and mediate the release of itch-inducing gastrin-releasing peptide and analgesic including endogenous opioids (Dawson and Karpen 2014). It is important to consider that microbes not only secrete metabolites but alter the availability of precursors that are essential for neuronal/gut homeostasis. Whether it is neurotransmitters, bile acids, or SCFAs, each are integral to maintaining gut health and function. Drug-mediated dysbiosis, or even small reductions/expansions of community members, appears to be enough to exacerbate opioid-related signaling, inflammation, and intestinal permeability.

### Opioid Use, Intestinal Permeability, and Inflammation

Opioid-induced gut microbial disruption and bile acid dysregulation (as have been noted in the previous sections) leads to gut barrier compromise and sustained systemic inflammation (Wang et al. 2018a). Individuals with heroin use disorder display distinct increases in gut microbiota diversity and composition compared with healthy controls (Xu et al. 2017). Opioid-use-dependent microbial dysbiosis is independent of liver disease, instead resulting in increased endotoxemia and hospital readmissions (Acharya et al. 2017). Morphine-treated animals also exhibited significant changes to secondary bile acid availability, which ensued after primary bile acids decrease following a reduction in *Lactobacillus* and *Clostridium* (Banerjee et al. 2016). Secondary bile acids have been implicated in gut barrier disruption and increased intestinal inflammation. Preclinical studies have also demonstrated impaired intestinal epithelial repair in humanized mouse models, which plays a crucial role in the overall immune response of the host (Meng et al. 2015b, 2020).

Clinical studies also find issues with gut epithelial integrity following opioid abuse. Opioid-induced loss of epithelial integrity increases the likelihood of bacterial translocation and expression of proinflammatory cytokines such as IL-1β in the colon. Opioid use was also associated with a 1.5-fold increased risk of mortality from colonic inflammation and a three-fold risk of infection when compared with patients not receiving opioid analgesics (Lichtenstein et al. 2012). Neutralization of IL-17A after morphine exposure improves intestinal barrier function in a sepsis model of mice (Meng et al. 2015a). Blocking intestinal barrier degradation stops the translocation of MAMPs such as LPS, is a common trigger of the immune system which has been linked to the development of anxiety and depression-like behaviors in mice, which can exacerbate drug-taking behavior Jang et al. (2019). In parallel with microbial-related inflammation, opioids also initiate a neuroinflammatory response within the CNS through toll-like receptor 4 (TLR4) microglia activation, which can increase tolerance and reduce opioid-induced analgesia (Milligan and Watkins 2009; Hutchinson et al. 2007). Ongoing pain and the negative emotional states related to opioid withdrawal are a significant incentive to continue or escalate opioid use (Koob 2020; Carmack et al. 2019). Alterations of pain via microbiome pathways may play an important role in the escalation of use following exposure. Indeed, antibiotic-induced depletion of the microbiome after morphine treatment reduced inflammatory mediators such as IL-1β, relating the importance of separating drug-related microbe inflammation and non-drug related inflammation (Kang et al. 2017). Inflammation of the colon has also been demonstrated to increase antinociceptive tolerance to morphine (Komla et al. 2019). Opioids induced activation of TLR4 signaling has also been associated with changes to morphine-tolerance and reward behaviors (Hutchinson et al. 2012; Wang et al. 2012a). Antagonism of the TLR4 pathways has been demonstrated to reverse neuropathic pain and to potentiate opioid analgesia (Hutchinson et al. 2012; Watkins et al. 2007). Lee et al. (2018a) hypothesized that systemic administration of opioids at given intervals, as well as cessation, would impact inflammation-driven hyperalgesia. They demonstrated a causal relationship between intermittent morphine exposure and dysbiosis and ensuing reward-related behavior. In the brain, increased microglia activation in the VTA leads to a reduction in dopamine-dependent reward behavior through a BDNF/microglia-mediated pathway (Taylor et al. 2016). While studies at the intersection of OUD and the gut-brain axis are still developing—there is promising evidence that the microbiome may play a significant role in harm reduction from opioid use. Opioids are unique in that the mechanism in which they would reduce pain also contributes to immune mechanisms that increase pain, a major incentive for dependent individuals to escalate and/or continue drug use. Alterations of the microbiome leading to gut metabolite shifts and increased inflammatory responses seem to be omnipresent following exposure to drugs of abuse. Decreasing inflammation broadly whether via the microbiome or microbiome related treatments appears to be a viable potential target to reduce comorbid psychiatric disorders and negative emotional/physical states that contribute to the modulation of drug taking behavior.

## Psychostimulants and the Microbiome

This section will focus on cocaine and methamphetamine, of which exposure and withdrawal have been demonstrated to impact the gut microbiota and induce depressive-like behavioral effects (Volpe et al. 2014). Depending on the population, prescription stimulants for the treatment of ADHD are also commonly abused. Stimulants act as an appetite suppressant, cause constipation, diarrhea, and acute intestinal ischemia leading to shifts in the resident microbiota, an increase in intestinal permeability, and inflammation, all of which contribute to comorbid stress, anxiety, and depression (Ersche et al. 2013). The significant influx of neurotransmitters in the gut-associated with stimulant usage can also lead to the generation of oxidative stress molecules which damage or kill enteric neurons, compounding the impact of stimulants on the gut (Yu et al. 2015). Increased neurotransmitter release, such as the kind found in stimulant use, can stimulate microbial blooms, which may impact the stress response (Freestone et al. 2002). Though the literature is limited, there are some notable foundational works demonstrating the modulation of drug-taking behavior through perturbations of the gut-brain axis.

### Stimulant Use and Microbes

Preclinical cocaine exposure in mice results in decreases the abundance of *Mucispirillum*, *Ruminococcaceae*, *Lachnospiracea*, and *Butyricicoccus,* with an increase in *Barnesiella, Porphyromonadaceae, Bacteriodales,* and inflammation inducing Proteobacteria (Scorza et al. 2019; Chivero et al. 2019; Ning et al. 2017). Similarly, rats exposed to methamphetamine also exhibit a reduction in SCFA producing microbes such as *Ruminococcaceae*, *Lachnospiracea*, and *Butyricicoccus* as well as related circulating metabolites (Ning et al. 2017). As observed with other drugs of abuse, cocaine reduces diversity and richness within the gut microbiome (Scorza et al. 2019). In contrast, methamphetamine-treated animals were reported to have increased diversity following exposure. While characteristic increases in Proteobacteria were observed following methamphetamine exposure, there was also an increase in *Ruminococcaceae*, which has also been reported to be reduced following methamphetamine use (Ning et al. 2017). In the clinic, human methamphetamine users demonstrate a reduction in beneficial SCFA producing *Butyricicoccus and Fecalibacterium* and an increase in pathogenic *Porphyromonas,* which is associated with periodontal disease, a common comorbidity in methamphetamine users (Cook et al. 2019). Stimulants elicit similar decreases in SCFA producers and increases in proinflammatory inducers observed in other substance use disorders such as AUD and OUD.

### Microbiome Manipulation Alters Substancse Use-Related Behaviors

Depletion of the microbiome by antibiotics has been demonstrated to affect stimulant taking behavior. Kiraly et al. demonstrated that depletion of the microbiome in animals exposed to cocaine lowered the reward threshold for conditioned place preference for cocaine, which returned to control levels using SCFA supplementation (Kiraly et al. 2016). Those animals also exhibited alterations of gene expression in the nucleus accumbens, a region essential for reward-related behaviors. Antibiotic depletion increased brain-derived neurotrophic factor (BDNF) and dopamine receptor type-1 (Drd1) and decreased neurotrophic receptor tyrosine kinase 2 (Ntrk2), demonstrating that depletion of the microbiome was associated with increased reward and sensitized behavioral responses to cocaine. This key finding was crucial for linking SCFA administration, microbiome manipulation, and behavioral changes to a drug of abuse. In contrast to these results, Lee et al. found that depletion of the microbiome decreases cocaine conditioned place preference (CPP) at a similar dose (Lee et al. 2018a). The differences are unsurprising; the two studies used different depletion protocols, and one had previous exposure to opioids which can impact microbial communities, as well as the neuronal ensembles activated during intoxication and withdrawal (Simpson et al. 2020). The differences between these studies are further discussed in the review by (Meckel and Kiraly 2019).

### Stimulant Use—Metabolites, Immune system, and Intestinal Permeability

Levels of SCFAs are associated with inflammatory responses. Exposure to stimulants can lead to significant disruption of gut function and signaling through the combination of a decrease in SCFA producers and the exhaustion of endogenous neurotransmitters (Ning et al. 2017). Like cocaine, alcohol, and opioids, methamphetamine increases susceptibility to infections through the alteration of immune activity (Cook et al. 2019). Many of methamphetamine’s neurotoxic effects are mediated by inflammation (Potula et al. 2018; Prakash et al. 2017) with exposure to the drug impacting both adaptive and innate immunity (Salamanca et al. 2014), modifying cytokine pathways, and inhibiting T-cell proliferation (Potula et al. 2018). Methamphetamine use increases IL-6 and IL-8 production, which stimulates inflammatory responses in the brain, inhibits neurogenesis, and alters hippocampal function and progenitor cell propagation (Salamanca et al. 2014). Methamphetamine also blocks proliferation of astrocytes by altering gene expression (Jackson et al. 2014; Shah et al. 2012). Methamphetamine users are more susceptible to infections, likely due to a decrease in macrophages, natural killer cells (NK), dendritic cells (DC), monocytes, and granulocytes seen following methamphetamine use (Harms et al. 2012; Saito et al. 2006). Cocaine was also found to induce a myriad of changes in levels of expression of cytokines (TNFa, IL-6, MCP-1), chemokines, and toll-like receptor activation (TLR-2) in vitro, ultimately leading to a proinflammatory environment (Liao et al. 2016). The largest increases are seen in cytokines MCP-1 and CCL-11, both of which are pro-inflammatory. Cocaine-mediated gut dysbiosis was also associated with the upregulation of proinflammatory mediators including NF-kB, and IL-1β (Chivero et al. 2019). Like in cocaine paradigms, mice treated with methamphetamine also exhibit increased expression of IL-1β in the brain (Loftis and Janowsky 2014).

A large proportion of metabolites in the blood originate in and are functionalized/deconjugated in the gut (SCFAs, Bile Acids). Therefore, it is reasonable to conclude that alterations of the resident microbiota may impact the normal interactions of the microbiome metabolites and cytokines, macrophages, NK, and DC’s (Sridharan et al. 2014; Krishnan et al. 2018). For instance, microbial tryptophan metabolism is known to modulate cytokine protection at the level of host metabolism Schirmer et al. (2016). Chivero et al. demonstrated that cocaine exposure in mice contributed to the degradation of the mucosal epithelial barrier composition and decreased integrity of the gut epithelium through the claudin family of proteins which regulate tight junction proteins, leading to an increase in intestinal permeability (Chivero et al. 2019). Aside from intestinal permeability, methamphetamine and cocaine exposure increases blood–brain barrier (BBB) permeability (Barr et al. 2020; Northrop and Yamamoto 2015). With the simultaneous increased release of bacterial metabolites and bacterial translocation combined with a compromised BBB, gut-derived metabolites can have a greater impact on brain signaling and inflammation. While these studies are noteworthy and highlight the importance of the microbiome at multiple levels of stimulant pathology, more mechanistic studies are needed to separate which perturbations have the greatest contribution to the related downstream psychostimulant abuse. Further study the ability of depletion microbiome to alter the reward threshold for stimulants may uncover microbes that can confer resistance to addiction liability.

## Nicotine and the Microbiome

Despite widespread use of nicotine, the literature on nicotine use and the gut microbiome is sparse compared to other drugs of abuse. Nicotine use, whether via cigarettes, vaping, or chewing, impacts the microbiome of the mouth, gut, and respiratory tract (Savin et al. 2018; Mason et al. 2015; Prochaska and Benowitz 2019). Smoking is a known risk factor for gastrointestinal cancers, Crohn’s disease, liver disease, and H. pylori infections (Li et al. 2014; Berkowitz et al. 2018). Like other drugs of abuse, tobacco use can contribute to negative impacts on the immune system (Jaspers 2014). Current and previous smokers have significantly reduced bacterial diversity in upper small intestinal mucosa compared to those who have never smoked. Shanahan et al. also observed that smokers exhibited a higher relative abundance of Firmicutes and Actinobacteria (*Rothia)*, with lower levels of Bacteroidetes (*Prevotella)* and Proteobacteria (*Neisseria)* (Shanahan et al. 2018). These results contrast with other studies where there appears to be an increase in Proteobacteria and Bacteroidetes, as well as *Clostridia* and *Prevotella,* but this is dependent on the route of administration (Capurso and Lahner 2017; Stewart et al. 2018; Benjamin et al. 2012). Vaping nicotine did not result in the same shifts as smoking tobacco via combustible cigarettes (Stewart et al. 2018). Yet another study reported a decrease in Actinobacteria and Firmicutes, as well as *Bifidobacterium* and *Lactococcus* (Biedermann et al. 2013). Moreover, the microbiome appears to go through a rebound stage following smoking cessation (Lee et al. 2018b). Nicotine has a wide range of methods of intake potentially supporting the observed variety in the changes to the resident microbiome than other drugs of abuse. For a further in-depth cross-sectional analysis of smoking, the oral/gut microbiome, and a wide range of comorbid diseases, see Huang and Shi (2019).

Vogtmann et al. (2015) investigated the effect of smoking on the upper gastrointestinal (UGI) microbiome and found that smoking was associated with both increased alpha and beta diversity, in contrast to the study done by Shanahan et al. (2018) in which they reported decreased diversity. Vogtmann also reported a greater abundance of two bacterial species in the UGI of smokers – *Dialister invisus* and *Megasphaera micronuciforms*. They theorized the gram-negative, anaerobic nature of these bacterium might give them an advantage in a smoky environment (Vogtmann et al. 2015). One theory they posed for the seemingly heightened diversity was the presence of different bacterial species in cigarettes. Several groups demonstrated that both cigarettes and smokeless tobacco products harbor diverse microbial populations both within samples and between different brands. Many of the microbes identified in these studies are opportunistic and capable of causing infections. (Vogtmann et al. 2015; Sapkota et al. 2010; Smyth et al. 2017). Another theory involves the immunosuppressive nature of tobacco, allowing for increased diversity (Vogtmann et al. 2015). It seems that study design, sampling location, cigarette preference, and population contribute to these contrasting results and should inform future study design.

Preclinically, nicotine has also been demonstrated to impact the microbiome differently in males and female mice. Chi et al. demonstrated sex-dependent effects of nicotine on gut microbiome community composition, functional bacterial genes, and fecal metabolome. Fecal metabolomics showed that neurotransmitters, such as glutamate, GABA, and glycine, were differentially altered in this model, translating to modification of gut signaling (Chi et al. 2017). Another study, also demonstrated changes in microbiome related metabolites following smoke exposure. In this study, rats exposed to nicotine exhibited decreased levels of SCFA (Tomoda et al. 2011). Recent studies have also demonstrated that cigarette smoking increases cholesterol in the liver and altered related bile acid metabolism, perhaps through microbiome modifications resulting from smoking (Yang et al. 2021). These observations yet again highlight that intake of drugs of abuse broadly alter microbiome composition and downstream metabolites that closely regulate immune and brain function. In addition to shifts in intestinal microbiota composition, smoking has also shapes bacterial makeup of the oral microbiome. Alterations in the oral microbiome were also observed in active methamphetamine users. Specifically, current smokers appear to have increased levels of Proteobacteria*, Atopobium, and Streptococcus,* all of which are linked to pathogenicity and inflammation (Wu et al. 2016). Smoking-induced oral microbiome dysbiosis also contributes to periodontitis and related downstream inflammation (Wade 2013). Furthermore, disruption of the oral microbiome directly effects the state of the gut microbiome (Olsen and Yamazaki 2019).

### Nicotine and the Immune System

There are relatively few studies examining the relationship between the microbiome, immune response, and smoking; however, smoking is known to alter the immune system by increasing the numbers of neutrophils, macrophages, eosinophils, and mast cells (Mehta et al. 2008). An expansion of these processes can result in immune suppression, which can leave the host at a higher risk of colonization of commensal and pathogenic bacteria (Droemann et al. 2005; Matthews et al. 2012). Chronic cigarette smoke exposure also induces microbial and inflammatory shifts as well as changes in the protective mucin layer in preclinical models (Allais et al. 2016). As seen in stimulants, opioids, and alcohol, IL-6 (Gellatly et al. 2020) and IL-8 (Johnson et al. 2010) are also both upregulated following exposure to nicotine. Smoking also impedes activation of NF-κB, a central immune system regulator. Suppression of NF-κB, in turn, reduces the gut inflammatory response (Wang et al. 2012b). Intestinal cytokine levels were also shown to be altered as a result of smoking with increased cytokine (MIP-2) and interferon (IFN-γ) levels and increasing IL-6, and decreasing TGF-β in the colon (Allais et al. 2016). Allais et al. offered evidence to implicate smoking in alterations of intestinal mucin composition (Allais et al. 2016). Notably, they found increased mRNA expression of mucin proteins mucin 2 (Muc2) and mucin 3 (Muc3) in the ileum and mucin 4 (Muc4) in the colon of smoke-exposed mice. In addition, smoking-related alterations of oxidative stress-related enzymes have been hypothesized to be involved in smoking-induced gut dysbiosis (Wang et al. 2012b). There is conflicting evidence as to how long the microbiome alterations last and what the long-term impact is on the cessation / relapse of smoking. With these inconsistent results, there is still much more to be explored. Study sizes were small and involved heterogeneity in the route of administration (smoking, electronic cigarette) and population; however, with the prevalence of nicotine use in the population it should be considered a comorbidity for future microbiome and behavioral studies. Additionally, the upregulation of similar inflammatory pathways and pro-inflammatory microbes are observed after nicotine use as in OUD and AUD.

## Other Drugs of Abuse and the Microbiome

### Psychedelics

Psychedelics have regained popularity as therapeutic agents for stress-related disorders. Psychedelics, which include LSD, psilocybin, and DMT, among others, are serotonergic drugs that bind to serotonin receptors, including 5-HT2A, which is known to be the pharmacological trigger of psychedelic experiences (Nichols 2016). Serotonin is a key neuromodulator involved in cognition, mood, and perception. Intake of psychedelics is also linked to suppression of feeding behavior. Chronic treatment with selective serotonin reuptake inhibitors remains the leading treatment for depression. Serotonin is widely produced in the gut, with up to 90% of the body’s supply being synthesized by enterochromaffin cells in the gastrointestinal tract (Hata et al. 2017; Yano et al. 2015). Approximately 50% of gut-derived Serotonin is regulated by the gut microbiota, dominated by *Clostridiaceae* and *Turcibacteraceae* (Fung et al. 2019; Reigstad et al. 2015). It is possible that serotonergic drugs not only interact with downstream receptors of the host but also on microbes that sense and metabolize serotonin (Fung et al. 2019). Lasting impacts to mood and behavior have been documented with micro-dosing, which do not have the characteristic central psychedelic responses but have been theorized to be driven through peripheral mechanisms, perhaps mediated by gut microbes or microbe related metabolite mechanisms (Kuypers 2019).

Psychedelics such as LSD appear to diverge from other drugs of abuse as they have reported anti-inflammatory properties. This has been demonstrated through the suppression of B-lymphocytes (2015) and NK cells as well as the suppression of the induction of IL-6, IL-4, and IL-2 in vitro (House et al. 1994). 5-HT2 agonism has also been demonstrated to reduce expression of proinflammatory markers (IL-6, TNFa, and others) in vivo (Flanagan et al. 2021). Serotonin can also modulate macrophage and dendritic cell function (de las Casas-Engel et al. 2013; Szabo et al. 2018). Whether alterations of neurotransmitters or immune status, there are few studise of psychedelics on the microbiome. The promising anti-inflammatory properties of psychedelics and the direct interaction with host serotonergic system make this class of drug appealing to study in conjunction with the microbiome to modify host behavior.

### Cannabis

Cannabis is a complex plant comprising over 400 chemical compounds, many of which have antibacterial properties (Appendino et al. 2008). Two of the most widely studied compounds are cannabidiol (CBD) and tetrahydrocannabidiol (THC) (Atakan 2012), which are ligands of the cannabinoid receptors. The psychoactive and gut effects of cannabis are well known and are mediated via the cannabinoid receptors 1 and 2 (CB_1_, CB_2_) (Gyires and Zadori 2016; Casajuana et al. 2018). CB_1_ is highly expressed in the intestinal epithelium, smooth muscle, the submucosal myenteric plexus, and the brain. CB_2_ is differentially expressed from CB_1_ and is mainly found in plasma cells, macrophages, and at much lower levels in the brain (Wright 2005; Roche and Finn 2010). CB_1_ activation is primarily mediated via the gut-brain axis with both central and peripheral actions. Like mu-opioid receptor activation, CB_1_ activation reduces gastrointestinal motility and hyperalgesia. CB_1_ also reduced gastric acid secretion and nausea but increases feeding and binge-like behaviors (Sanger 2007). Recreational cannabis use is associated with a 30% reduction in constipation (Adejumo et al. 2019). CB_2_ activation functions mainly through the immune system and is hypothesized to be a gastrointestinal inflammation “braking mechanism” to mediate intestinal inflammation and limit visceral pain (Wright et al. 2008). The endogenous cannabinoid system has been demonstrated to protect against peripheral colonic inflammation (Massa et al. 2004). Recent review has thoroughly examined the role of CBD and the immune system and reports that CBD is immune suppressive in both in vitro and in vivo models (Nichols and Kaplan 2019). In contrast, when whole cannabis was explored in individuals with cannabis use disorder, IL-1β, IL-6, IL-8 and TNF⍺ were increased in cannabis users compared to controls (Bayazit et al. 2017); however, immune modulation seems to be dependent on administration and model (Nagarkatti et al. 2009). Despite the apparent connection between cannabis and the microbiome, there have been few studies on the direct impact of cannabis and the microbiome/behavior.

Activation of CB_1_ has been demonstrated to acutely regulate gut epithelial barriers in a model of acute stress (Zoppi et al. 2011), and to modulate the activity of vagal neurotransmission in relation to gastrointestinal function (Vianna et al. 2012; Izzo and Sharkey 2010). Endocannabinoid control of feeding has been established by several groups (Gomez et al. 2002), and fasting is known to increase levels of anandamide in the rat small intestine (Gomez et al. 2002). Cluny et al. hypothesized that exposure to THC might produce weight loss due to the regulation of adipogenesis through endocannabinoid signaling, and therefore treated mice chronically with THC. THC blocked an increase in the Firmicutes:Bacteriodetes ratio in diet-induced obese mice and increased *Akkermansia Muciniphilia* spp. compared to lean controls. THC administration prevented weight gain in diet-induced obese mice with no significant effects on locomotor activity or energy intake. Further investigation is needed to determine if the effects are reciprocal and additive (Cluny et al. 2015). Comparable to the previous study, hedonic eating in humans is associated with increases in endocannabinoid signaling. Monteleone et al. demonstrated increased peripheral levels of endocannabinoid, 2-Arachidonoylglycerol (2AG), and ghrelin are generated by the availability of highly palatable food and pleasurable eating (Monteleone et al. 2012). Drugs of abuse are often initiated due to the rewarding effects of substance intake (Koob and Moal 2001), but are continued due to the emergence of negative emotional states when the drug is not available (Koob and Volkow 2016). Again, the literature is sparse for psychedelics and CBD but interest in their role in gut motility, permeability, and interactions with the gut microbiota is growing (DiPatrizio 2016). Expanding such studies will be essential in unraveling the contribution of different drugs to microbiome modification. Polydrug abuse is common, and if the microbiome is to be leveraged to improve substance use disorder outcomes, more work is needed.

## Discussion

This review aims to consolidate the current understanding of the bidirectional relationship between substance-use disorders and the microbiome and encourage future studies in this emerging cross-sectional field. Our analysis was concentrated on the impact that a wide range of substances have on the microbes, metabolites, and immunological profile of the host, and in turn, how those perturbations might alter drug-taking behavior.

**Fig. 2:**
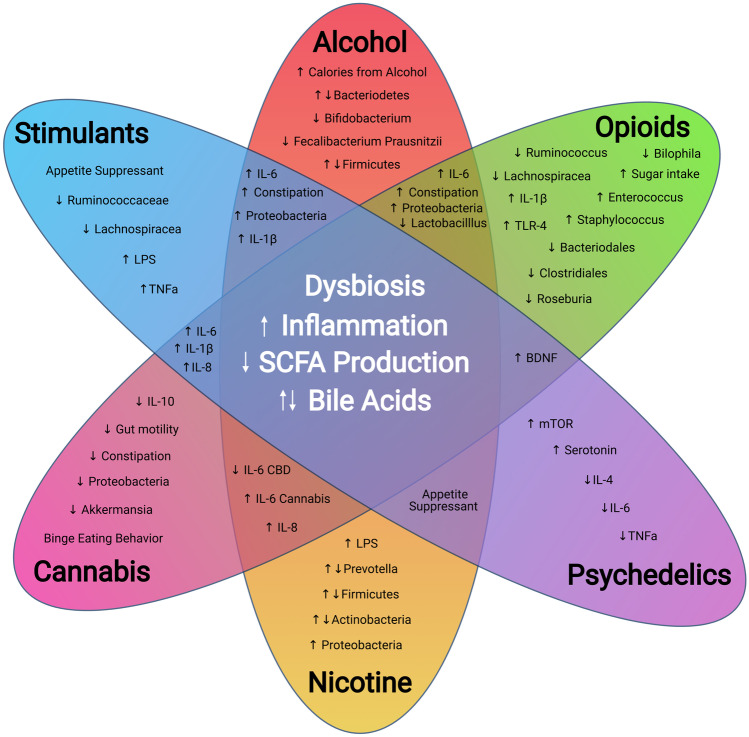
**Microbiome, Immune, and Metabolite Alterations Following Drug Exposure.** This diagram highlights the most prominent factors associated with the microbiome and drugs of abuse. Dysbiosis, increased inflammation and inflammatory microbes, a decrease in SCFA producing bacteria and SCFA production, along with bile acid dysregulation are shared among the assessed drugs. Constipation occurs among stimulants, alcohol, and opioids. Upregulation of IL-6 is conserved among almost all drugs except for cannabis and psychedelics. Appetite suppression, increased binge eating, and/or shifts in caloric sources are a common theme among all drugs surveyed. Despite varying mechanisms of action, drugs of abuse contribute to alteration of the microbiome that result in downstream immunological and metabolic shifts that in turn exacerbate drug related comorbidities and drug taking behavior. The microbiome remains a largely uncharted landscape for the development and discovery of novel therapies and biomarkers for substance use disorders. Abbreviations: BDNF (brain derived neurotrophic factor), CBD (cannabidiol), LPS (lipopolysaccharide), IL-4 (interleukin 4), IL-6 (interleukin-6), IL-8 (interleukin-8), IL-1β (interleukin 1β), mTOR (mechanistic target of rapamycin), TNF⍺ (Tumor necrosis factor ⍺), TLR-4 (toll-like receptor-4), SCFA (short-chain fatty acid)

Disturbance of the microbiome by drug exposure, dietary changes, and stress response all alter the epithelial layer in the gut. These barriers serve to protect the body from translocation of pathogenic microbes and microbial metabolites that lead to inflammation and increased inflammatory responses. Peripheral inflammation has been demonstrated across several drug groups and is associated with dysbiosis and increased adverse outcomes with the exception of limited reports of suppression of the immune system by psychedelics, cannabis, and nicotine; however, broadly, exposure to drugs alters the composition of the gut microbiota, production of microbial metabolites and immune status. These metabolite, microbial, and immunological changes following drug exposure are presented in Fig. 2. Preclinical investigations of microbiome manipulation of drug-taking behavior (Table 1) demonstrate deviations in reward, stress, and withdrawal responses. These drug-related microbiome alterations elucidate pathways (inflammation, SCFA, bile acid) that have the potential to alter central mechanisms underlying addiction vulnerability.

While a connection between the gut microbiome and substance use disorders is clear, there remains much to be explored – one critical concern being the frequency of polydrug abuse. For example, alcohol abuse is often accompanied by the use of nicotine, cannabis, and/or opioids. This renders the compounding effects of multiple drugs on microbiota an extremely relevant area of research for therapeutic development. Additionally, longitudinal studies investigating the microbiome-substance use disorder connection are relatively non-existent, and temporal effects of drug-use on gut microbiota and vice versa have yet to be elucidated. Drug use is often associated with comorbid disorders such as depression, stress, and anxiety, which are poorly documented in the current microbiome-addiction literature. While it may be difficult to tease apart the effect of medical and comorbid psychiatric conditions that coincide with substance use disorders on the microbiome, sequencing costs are declining and studies are increasing. Leveraging machine learning and available datasets will enable for better stratification, meta-analysis, and interpretation of the markers for each drug of abuse and between drugs of abuse. In addition, large scale GWAS studies and functional outputs such as metabolomics will help illustrate a more comprehensive picture of how the microbiome regulates human health. The potential to leverage the microbiome as a predictive tool to find novel pathways that contribute to abuse liability is in its nascency. Microbiome research is an evolving, multifaceted field that will require collaboration and standardization to improve implementation of translational measures capable of combatting substance use disorders. There is abundant potential to elucidate novel drug targets and therapeutics associated with the microbiome and related pathways.
